# Correlation of Morphology and Metabolism of Reproductive Traits in the Genus *Phrynocephalus* Around the Qinghai‐Tibetan Plateau

**DOI:** 10.1002/ece3.72029

**Published:** 2025-08-25

**Authors:** Xiaqiu Tao, Fan Xie, Lin Zhu, Qiannian Wu

**Affiliations:** ^1^ Jiangsu Key Laboratory for Biodiversity and Biotechnology, College of Life Sciences Nanjing Normal University Nanjing China; ^2^ Kunming Institute of Eco‐Environmental Science Kunming China

**Keywords:** morphological trait, offspring mass, reproductive output, size‐number trade‐off, standard metabolic rate

## Abstract

The trade‐off between offspring size and number is a crucial concept in life‐history theory, offering key insights into animal reproductive strategies. Our study examines the relationship between reproductive characteristics, morphological traits, and metabolism in a total of 290 female *Phrynocephalus* lizards across 10 species. Reproductive, morphological, and metabolic traits were compared by analyzing *Phrynocephalus* lizard species with viviparous and oviparous reproductive modes. The results show no significant differences in reproductive traits between oviparous (6) and viviparous (4) species. Snout‐vent length and standard metabolic rate positively correlated with offspring mass, while no correlation was found with offspring number. The lack of a trade‐off between offspring size and number indicates that larger females invest more in offspring mass rather than offspring number. These results, at least in this genus, were inconsistent with the classic prediction that females give priority to adjusting the number rather than the size of their offspring, enabling us to understand the evolution of the reproductive strategy in reptiles.

## Graphical abstract

We explored the correlation of maternal morphology, metabolism, and reproductive traits in 10 Phrynocephalus lizards around the Qinghai‐Tibetan Plateau. We found that oviparous and viviparous lizards did not differ in their reproductive traits. Phrynocephalus lizards with greater snout‐vent length and higher standard metabolic rates tended to produce heavier offspring instead of more offspring. Our study does not follow the prediction that reproductive females give priority to adjusting the number rather than the size of their offspring.

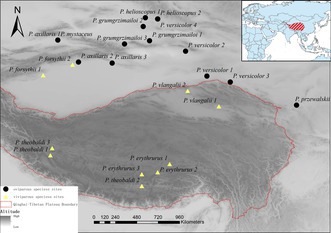

## 1 Introduction

The trade‐off between offspring number and mass is a crucial component in life‐history traits. Organisms face limited resource allocation, so an increase in one trait's advantage, such as offspring size, leads to a reduction in another, such as offspring number. This interplay plays a vital role in understanding animal reproductive output and the microevolutionary fitness (Ferguson and Fox 1984; Williams 1994; Räsänen et al. 2008; Meiri et al. 2020). Energy obtained in animals needs to be converted into usable energy forms. This is balanced between the demands of maintenance and production (Kubička and Kratochvíl 2009; Luo et al. 2010). Energy expenditure for maintenance encompasses basal (endotherms) or standard (ectotherms) metabolic costs, as well as the energy required for essential activities such as foraging, predator avoidance, and thermoregulation. Production (tissue growth, reproduction, and energy storage in descending order of priority) can be allocated after the demands for maintenance have been met (McNab 2002; Glazier 2009; Sibly et al. 2013). Reproduction is not essential for individual survival, but it is strongly linked to viability because the continuity of populations or species relies on individual reproduction (Sibly et al. 2012). When energy availability is limited, reproductive energy allocation typically decreases or may cease altogether (McNab 2002). When maternal reproductive investment decreases, trade‐offs may arise that lead to variations in reproductive traits such as egg‐laying intervals, offspring number, and offspring mass (Smith and Fretwell 1974; Charnov and Downhower 1995; Bja and Reznick 2011).

According to the classical trade‐off between the number and mass of offspring, variations in reproductive investments are expected to result in changes in offspring number rather than mass (Smith and Fretwell 1974). However, contrary to this prediction, empirical evidence from various animal taxa suggests that offspring mass can vary in response to changes in total reproductive investment, fecundity, and maternal size or body condition, sometimes without exhibiting a significant trade‐off (lizards: Bleu et al. 2013; snakes: Ford and Seigel 2015; Guo et al. 2022). Climate conditions, predation risk, availability of resources, and other environmental factors can also influence female reproduction. For example, it has been reported that food availability can regulate offspring number by affecting maternal body condition (Seigel and Ford 1992); predation risk shapes offspring number and investment in lizards (Foufopoulos et al. 2023); and cold climates are linked to reproductive mode evolution and thermoregulatory adaptations (Cruz et al. 2022).

There are several hypotheses that attempt to explain the relationship between the maternal trait and offspring size–number trade‐off in ectotherms. For example, (1) transition from oviparity to viviparity: viviparous animals may carry offspring for longer and experience greater costs than do similar oviparous ones (Qualls and Shine 1998); (2) morphological constraints: different pelvic aperture widths and caudal gap heights are attributed to different female sizes (Clark et al. 2001); (3) influence of maternal behaviors: behaviors such as prevention of predation would provide a more comfortable environment and enough nutrition for the offspring (Mitchell et al. 2015; Alarcón‐Ríos et al. 2024); and (4) physiological allocations: with increased energy allocation to reproduction, females can optimize their reproductive strategy by either enhancing investment in offspring number or size (Yanagi and Tuda 2012).

The genus *Phrynocephalus* (Agamidae) consists of approximately 38 lizard species (32 oviparous, 6 viviparous), exhibiting either oviparous or viviparous reproductive strategies (Barabanov and Ananjeva 2007). They are distributed in the deserts, semideserts, or sandy regions of Central Asia and Western Asia, including the Qinghai‐Tibet Plateau and the Mongolian Plateau. The Qinghai‐Tibet Plateau, with a mean altitude of over 4000 m.a.s.l. and an oxygen concentration from 20.12% to 20.72% (Hu et al. 2023), provides an ideal environment for studying the energy utilization and reproductive strategies of the genus *Phrynocephalus*, as the extreme conditions of high altitude and low oxygen levels have likely driven unique evolutionary adaptations. The uplift of the Qinghai‐Tibet Plateau, initiated roughly 40 million years ago by the collision of the Indian and Eurasian tectonic plates, created unique ecological niches that drove adaptive radiation and biodiversity; it is crucial for studying species evolution (Favre et al. 2015).

The genus is notable for its adaptation to the cold arid regions, especially in the vicinity of the Qinghai‐Tibetan Plateau. Their distribution range spans elevations from −40 m to 5300 m a.s.l. around this plateau. Viviparous *Phrynocephalus* species are endemic to China and can be found in the Qinghai‐Tibet Plateau and adjacent regions. *P. guinanensis*, *P. putjatia*, and *P. vlangalii* are found in the eastern part of the distribution range, while *P. erythrurus* , *P. forsythii* , and *P. theobaldi* are distributed further west, northwest, and southwest (Ji et al. 2009; Jin et al. 2014). Six species of oviparous *Phrynocephalus* can be found at lower altitudes around the Qinghai‐Tibetan Plateau, which are *P. axillaris* , *P. grumgrizimailoi*, *P. helioscopus* , *P. mystaceus* , *P. przewalskii* , and *P. versicolor* (Jin et al. 2018).

The average distribution altitude for viviparous *Phrynocephalus* lizards is around 3300 m a.s.l., which is higher compared to the oviparous species, averaging approximately 1200 m a.s.l. The lowest distribution altitude of the viviparous species is found as low as 600 m for *P. forsythii* . Conversely, the highest distribution altitude among the oviparous species is observed in *P. axillaris* at 3100 m (Zhao et al. 1999).

Most species possess extensive distribution ranges, marked by significant environmental heterogeneity within their respective territories. Each species has distinct distribution centers that differ from those of other conspecific species although there is often overlap in the distribution ranges.

The *Phrynocephalus* lizards exhibit significant differences in reproductive modes and physiological adaptations. Therefore, investigating the relationship between their reproductive strategies, morphological traits, and metabolic rates is essential for understanding their survival strategies in extreme environments. This study's main objective was to assess how different reproductive modes (oviparous and viviparous) in *Phrynocephalus* lizards from the Qinghai–Tibetan Plateau correlate with their metabolic rates and morphological characteristics. Additionally, we analyzed female reproductive traits and the influence of maternal condition on offspring size and number to evaluate the hypothesis of optimal offspring size theory.

## 2 Materials and Methods

### 2.1 Data Collection

**FIGURE 1:**
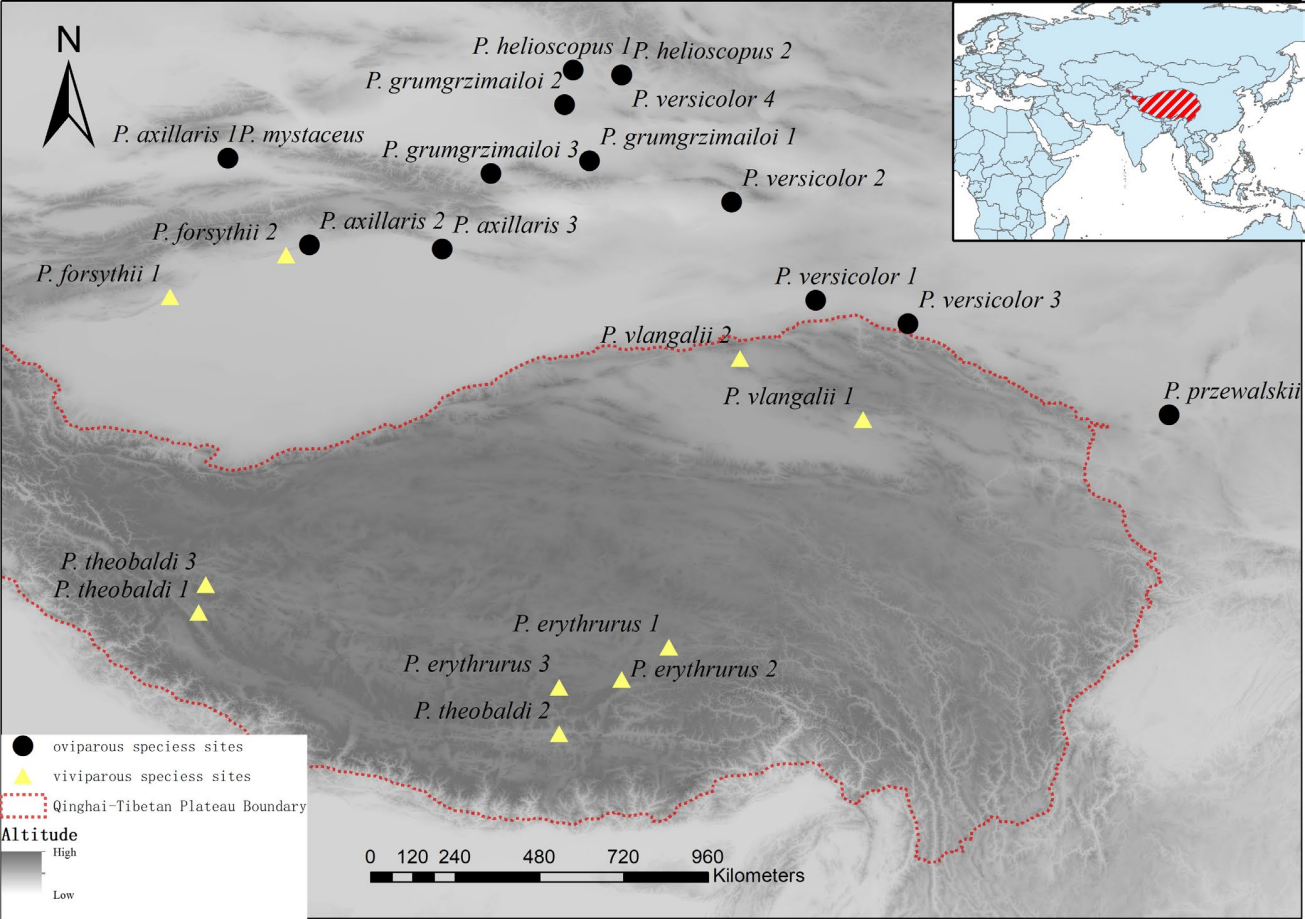
The sample sites of *Phrynocephalus* spp. The sites are denoted by yellow triangles (viviparous lizards) and black circles (oviparous lizards), the red dashed line indicates the approximate boundary of the Tibetan Plateau.

The *Phrynocephalus* spp. lizards used in this experiment were gravid adult females collected from the Qinghai‐Tibetan Plateau and adjacent regions between late April and mid‐July from 2019 to 2021 (Figure 1). The study included a total of 10 species (Table 1).

**TABLE 1:** Descriptive statistics, expressed as mean ± SE and range, for phenotype (SVL, AL, HL, HW, SL, SW, FLL, HLL), metabolic rate (SMR, mSMR) of newly hatched (oviparous species, O) and newborn (viviparous species, V) lizards.

Species	*N*	Mode	SVL	AL	HL	HW	SL	SW	FLL	HLL	CLS	CLM	ENM	RCLM	SMR	mSMR
			(mm)	(mm)	(mm)	(mm)	(mm)	(mm)	(mm)	(mm)		(g)	(g)			
*P. axillaris*	24	O	46.53 ± 0.56	22.35 ± 0.41	11.56 ± 0.17	10.63 ± 0.14	6.12 ± 0.11	9.80 ± 0.15	13.06 ± 0.26	18.48 ± 0.26	2.42 ± 0.22	1.14 ± 0.16	0.34 ± 0.05	0.52 ± 0.03	0.37 ± 0.06	0.11 ± 0.02
*P. erythrurus*	47	V	54.49 ± 0.45	28.04 ± 0.46	12.25 ± 0.09	10.99 ± 0.08	6.18 ± 0.08	10.04 ± 0.07	13.21 ± 0.15	17.28 ± 0.16	2.21 ± 0.11	1.79 ± 0.10	0.34 ± 0.02	0.83 ± 0.02	0.42 ± 0.05	0.08 ± 0.01
*P. forsythii*	32	V	47.41 ± 0.76	24.59 ± 0.53	11.14 ± 0.14	10.30 ± 0.11	5.45 ± 0.09	9.48 ± 0.11	12.55 ± 0.17	16.94 ± 0.28	3.56 ± 0.17	2.01 ± 0.12	0.50 ± 0.04	0.58 ± 0.02	0.48 ± 0.10	0.12 ± 0.02
*P. grumgrzimailoi*	39	O	54.02 ± 0.72	26.48 ± 0.52	13.09 ± 0.19	12.17 ± 0.18	6.78 ± 0.12	11.46 ± 0.29	15.11 ± 0.26	21.56 ± 0.26	2.18 ± 0.21	1.25 ± 0.19	0.18 ± 0.03	0.62 ± 0.03	0.78 ± 0.47	0.18 ± 0.13
*P. helioscopus*	8	O	46.03 ± 1.96	21.73 ± 1.11	11.70 ± 0.44	11.09 ± 0.38	6.21 ± 0.42	9.79 ± 0.55	14.12 ± 0.47	19.30 ± 0.39	3.00 ± 0.33	1.41 ± 0.12	0.33 ± 0.07	0.46 ± 0.05	0.87 ± 0.22	0.29 ± 0.08
*P. mystaceus*	5	O	75.38 ± 0.87	39.59 ± 1.15	19.06 ± 0.43	19.15 ± 0.28	11.71 ± 0.31	17.65 ± 0.38	25.85 ± 0.87	34.67 ± 0.79	3.00 ± 0.32	5.92 ± 0.62	0.35 ± 0.07	1.82 ± 0.15	1.13 ± 0.30	0.07 ± 0.02
*P. przewalskii*	9	O	51.57 ± 0.89	25.94 ± 0.48	13.26 ± 0.25	12.04 ± 0.23	6.82 ± 0.19	10.93 ± 0.25	14.77 ± 0.18	21.15 ± 0.21	1.22 ± 0.15	0.96 ± 0.10	0.16 ± 0.02	0.75 ± 0.06	——	——
*P. theobaldi*	41	V	47.58 ± 0.56	25.17 ± 0.41	11.44 ± 0.13	10.05 ± 0.10	6.02 ± 0.09	8.99 ± 0.10	12.56 ± 0.16	16.71 ± 0.18	2.32 ± 0.12	1.49 ± 0.10	0.36 ± 0.03	0.65 ± 0.02	0.29 ± 0.04	0.07 ± 0.01
*P. versicolor*	64	O	46.91 ± 0.35	22.76 ± 0.27	11.45 ± 0.09	10.53 ± 0.08	6.01 ± 0.06	9.58 ± 0.07	12.81 ± 0.14	18.34 ± 0.18	2.28 ± 0.15	1.12 ± 0.09	0.30 ± 0.03	0.49 ± 0.02	0.34 ± 0.06	0.11 ± 0.03
*P. vlangalii*	21	V	63.23 ± 0.91	34.23 ± 1.00	14.43 ± 0.18	12.51 ± 0.15	7.73 ± 0.13	11.08 ± 0.14	16.41 ± 0.21	21.77 ± 0.28	2.67 ± 0.21	2.54 ± 0.27	0.20 ± 0.01	0.92 ± 0.05	——	——

For each captured individual, the following morphometric variables were recorded: snout‐vent length (SVL, from the tip of the snout to the anterior edge of the cloacal vent), head length (HL, from the tip of the snout to the posterior edge of the ear opening or the concealed tympanum), abdomen length (AL, from the axilla of the forelimb to the groin of the hindlimb), head width (HW, linear distance across the widest part of the head), snout length (SL, from the tip of the snout to the posterior edge of the last supralabial scale), snout width (SW, linear distance across the snout at its widest point), fore‐limb length (FLL, from the axilla to the tip of the longest digit, excluding the claw), hind‐limb length (HLL, from the base of the hindlimb to the tip of the longest toe, excluding the claw). To minimize experimental errors, we used the same caliper with an accuracy of 0.01 mm for measurements and had the same experimenter perform the operations and readings of the data.

All individuals (*n* = 290) were housed under laboratory conditions in Nanjing, Jiangsu province (E118.91125; N32.10296), and kept in 530 × 400 × 310 mm (length × width × height) plastic cages. The cages contained a sand substrate and pieces of clay tiles for cover. The cages were placed in a rearing room equipped with an automatic air conditioning system. The system would turn on when the temperature reached 30°C. The density of lizards in each cage was under 8 individuals. They were provided with an ample supply of small‐sized mealworms, pinhead crickets, and water enriched with vitamins and minerals. We use clutch or litter size (CLS, the number of all eggs or neonates produced by a single female in a nest), clutch or litter mass (CLM, the total fresh mass of all eggs or neonates produced by a single female in a nest), egg or neonate mass (ENM, the average fresh mass of single egg or neonate), and relative clutch or litter mass (RCLM, the ratio of clutch/litter mass to postpartum female body mass) to describe reproductive characteristics.

During the breeding season, cages were inspected at least once every hour during the morning, as oviposition typically occurred early in the day. Upon discovery of a clutch or litter, we immediately weighed and measured all eggs or neonates within 6 h of birth (Wang et al. 2013). Water was provided via shallow dishes, and sand substrate was moistened twice daily to maintain humidity and prevent egg desiccation (Wang et al. 2017). Some studies showed that the eggs laid by squamates weighed the same as the hatchlings that emerge from those eggs several weeks later (Meiri et al. 2020).

After the breeding season, standard metabolic rate (SMR) and mass‐specific standard metabolic rate (mSMR) of maternal lizards was measured by the multiplexed respirometry systems (Sable Systems, Las Vegas, NV, USA). The SMR measurement was performed at a controlled temperature of 30°C. Each lizard was acclimated within the chamber of the circuit in an incubator at test temperatures for 30 min. During the acclimation, the chamber was flushed with dry air at a flow rate of 100 mL/min. After a temperature acclimation period of 30 min, the circuit system was sealed, and the rate of carbon dioxide production ($\dot{V}{\mathrm{CO}}_{2}$) in the system was continuously recorded secondly. The record length was set as 15 mins. All lizards were resting without any activity under the dark condition in the chamber during measurements. To minimize the impact of food digestion, the experimental animals underwent a 2‐day fasting period prior to the trials started. To mitigate the influence of diurnal rhythm, experiments were conducted between 7:00 and 17:00 with a quiet experimental environment. Carbon dioxide production was used for metabolic rate calculation. After measurement, lizards were released back to the cages (Sun et al. 2022). After the experiments, all hatched or produced offspring, along with the adults that completed the measurements, were released back to their original sampling sites.

### 2.2 Phylogenetic Analysis

Based on previous studies and our own field work in recent years, we compiled 10 published mtDNA cytochrome *b* (cyt *b*) genes (GenBank IDs AY053901, AY053906, AY053915, AY053936, AY053946, AY053967, AY053976, AY053991, KF691627, KF691632) and NADH dehydrogenase subunit 2 (*ND2*) (GenBank IDs: KC551441, KC551466, KC551478, KC551447, KC551438, KC551435, KC551448, KC551459, KC551443, KC551474), as well as the nuclear gene recombination activating gene 1 (*RAG1*) (GenBank IDs: KC551399, KC551423, KC551432, KC551405, KC551396, KC551393, KC551406, KC551417, KC551401, KC551428). To construct a phylogeny for *Phrynocephalus* clades, we concatenated the three gene regions for each species using ACOPTools and aligned them with concatenated sequences of the outgroup species *Paralaudakia lehmanni* (GenBank IDs: KF691618, GQ242229, KJ363482).

**FIGURE 2:**
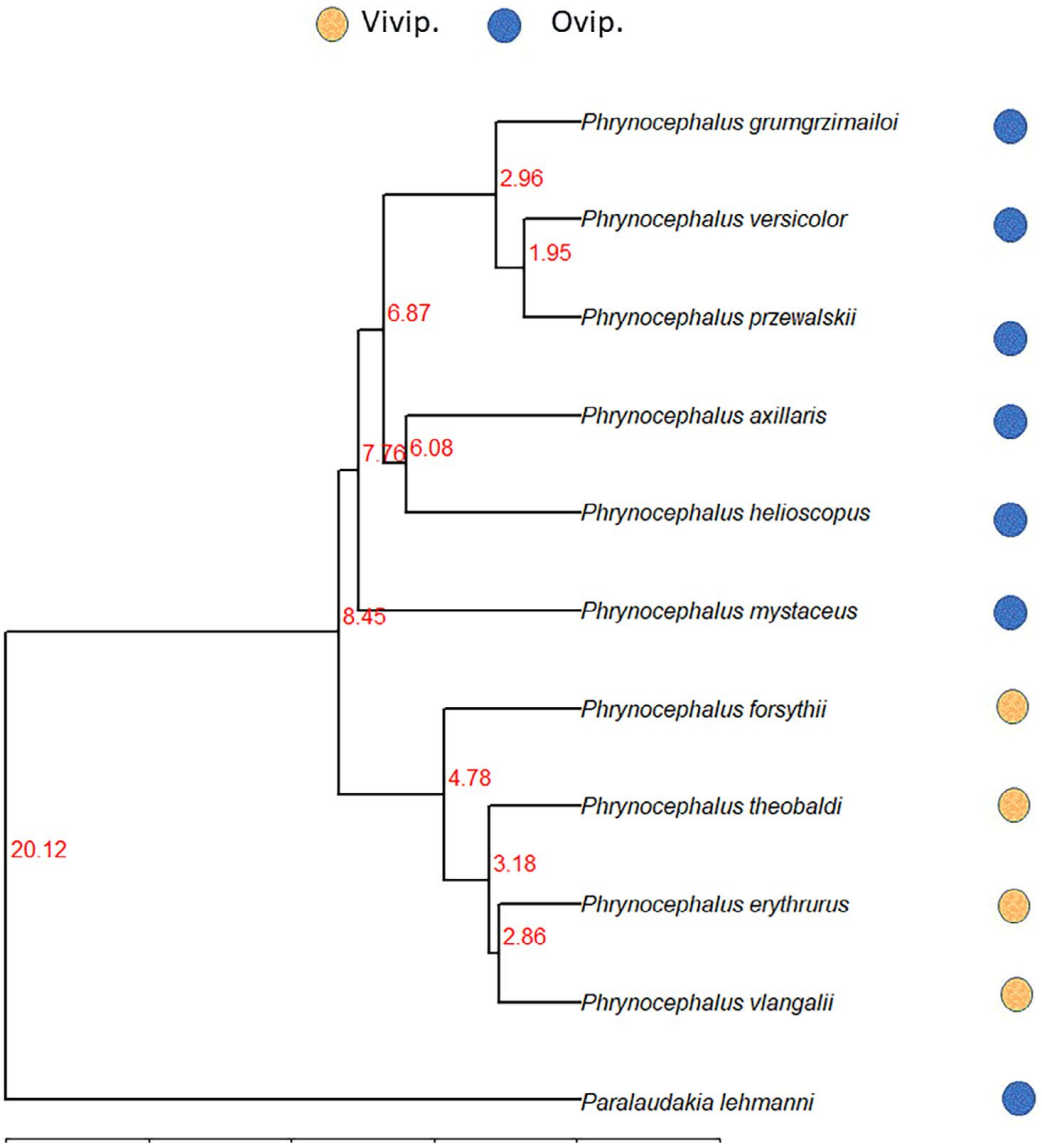
The phylogeny of the 11 species of lizards obtained from maximum likelihood analysis based on mitochondrial and nuclear genes. Numbers on each node of the tree indicate divergence time (millions of years ago, ma).

Because there are currently no fossil records for *Phrynocephalus* species within China, we used a secondary calibration point of (9.12 ± 1.44 Ma) for the divergence between viviparous and oviparous clades of *Phrynocephalus* (Guo and Wang 2007). Phylogenetic trees were constructed separately by using maximum likelihood (ML), implemented in PAUP v4.0a169 (Figure 2; Swofford 2003).

### 2.3 Statistical Analysis

All statistical analyses were performed with R 4.3.1 (R Development Core Team 2023). Phylogenetic generalized least squares (PGLS) regression and phylogenetic analysis of variance (phyANOVA) were conducted using caper 1.0.3 (Orme et al. 2023) and phytools 2.0‐3 (Revell 2024) to analyze the correlations between phenotypic traits, metabolism, and reproductive traits. We standardized all phenotypic data by dividing each trait by snout‐vent length (SVL) to remove the influence of individual size, except for snout length and snout width, which were corrected using head length. To mitigate collinearity in phenotypic data, PCA was conducted on the morphological traits related to head size and limb length. Separate principal components were extracted to represent head size and limb length. We used CorMatrix to perform the phyPCA. PGLS was conducted to account for the nonindependence of data due to the shared evolutionary history among species. Model selection was performed using the corrected Akaike Information Criterion (AICc). The strength of phylogenetic signal in phenotypic traits was indicated by Pagel's (1999) lambda (*λ*). If *λ* = 0, it suggests no significant correlation with phylogeny; if 0 < *λ* < 1, it indicates some degree of correlation with phylogeny; if *λ* = 1, it signifies a strong association with phylogeny.

## 3 Results

**FIGURE 3:**
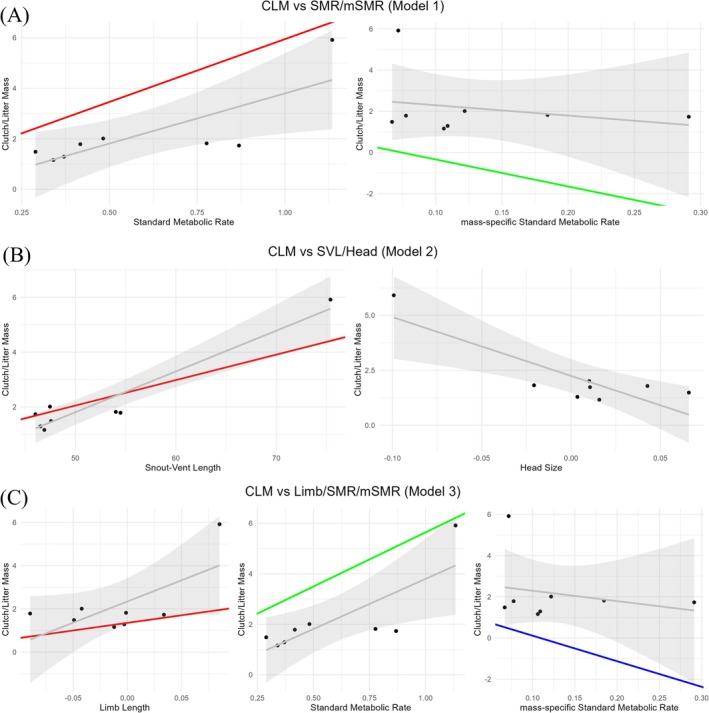
The phylogenetic generalized least squares (PGLS) analyses of the relationship between clutch/litter mass (CLM) and metabolic/morphological traits in *Phrynocephalus* Lizards. (A) CLM versus SMR/mSMR; (B) CLM versus SVL/Head; (C) CLM versus Limb/SMR/mSMR. The red/green/blue straight line represents the PGLS regression slope. The curved shaded area represents the 95% confidence interval.

The PGLS analysis showed that CLM exhibited a strong phylogenetic signal (*λ* = 1.19, *p* = 0.019). For CLM, models including metabolic rates, SVL, and head showed significant effects. In the best model (model 1), both SMR (slope = 4.98, *p* < 0.001) and mSMR (slope = −13.11, *p* = 0.002) were significant (*F*_(2,5)_ = 44.200, *p* < 0.001, *r* ^*2*^ = 0.9465). In the second‐best model (model 2), SVL (slope = 0.09, *p* < 0.001) and head (slope = −12.57, *p* < 0.05) were significant (*F*_(2,7)_ = 28.200, *p* < 0.001, *r* ^*2*^ = 0.8895). In the third‐best model (model 3), adding limb retained significance (*F*_(3,4)_ = 40.870, *p* = 0.002, *r* ^*2*^ = 0.9684) for SMR (slope = 4.29, *p* < 0.001) and mSMR (slope = −12.40, *p* < 0.001) but not limb (slope = 7.05, *p* = 0.17) (Figure 3).

For ENM, the best model (model 1) indicated SVL (slope = 0.034, *p* < 0.001) and limb (slope = 1.98, *p* = 0.027) were significant (*F*_(2,7)_ = 60.810, *p* < 0.001, *r* ^*2*^ = 0.9456).

In contrast, for CLS and RCLM, none of the models showed statistically significant relationships with the tested predictors (all *p* > 0.05) (Table 2).

**TABLE 2:** Phylogenetic generalized least squares (PGLS) analysis of *Phrynocephalus* reproductive traits.

Model ID	Model	*λ*	*r* ^2^	*F*	*p*	AIC_C_
CLS 1	SMR	0	0.152	1.070	0.340	15.787
CLS 2	mSMR	0	0506	0.320	0.592	16.687
CLS 3	AL, mSMR	0	0.321	1.180	0.380	19.604
CLM 1	SMR^**a**^, mSMR^**b**^	1	0.947	44.200	< 0.001	16.939
CLM 2	SVL^**b**^, head^**c**^	1	0.890	28.200	< 0.001	20.598
CLM 3	Limb, SMR^**b**^, mSMR^**b**^	1	0.968	40.900	0.002	22.054
ENM 1	SVL^**a**^, limb^**c**^	0	0.946	60.800	< 0.001	−11.329
ENM 2	SMR^**a**^, mSMR^**a**^	0	0.937	98.000	< 0.001	−9.789
ENM 3	SVL^**a**^, head^**c**^	0	0.975	51.600	< 0.001	−9.632
RCLM 1	mSMR	0	0.020	0.124	0.7371	−14.478
RCLM 2	SMR	0	0.006	0.037	0.853	−14.364
RCLM 3	SVL	0	0.100	0.886	0.3744	−14.329

^a^ Represents a significance level of 0.001.

^b^ Represents a significance level of 0.01.

^c^ Represents a significance level of 0.05.

**FIGURE 4:**
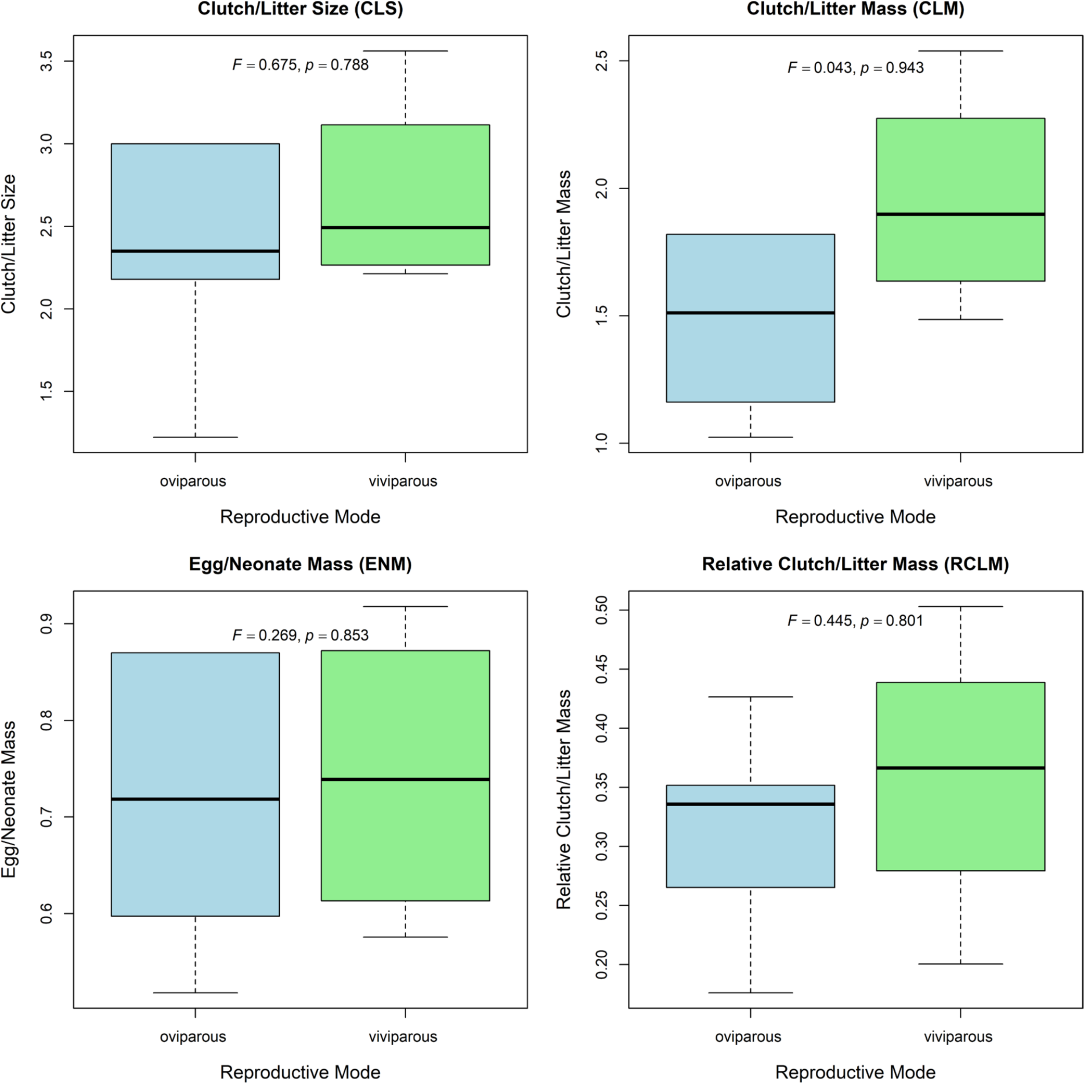
Box plot for comparison of reproductive traits between oviparous and viviparous Phrynocephalus species. *X* axis: Reproductive mode (oviparous and viviparous). *Y* axis: Reproductive traits. *F*‐value and *p*‐value on the boxplot are extracted from the results of phylANOVA.

The results of the phyANOVA showed no differences between the two reproductive mode groups (CLS: *F* = 0.675, *p* = 0.788; CLM: *F* = 0.043, *p* = 0.943; ENM: *F* = 0.269, *p* = 0.853; RCLM: *F* = 0.445, *p* = 0.801) (Figure 4). Partial correlation analysis revealed that the relationship between offspring size (CLS) and mass (ENM) was nonsignificant in each lizard species, with predominant correlations observed after controlling for maternal SVL (Table 3; all *p* > 0.05).

**TABLE 3:** Partial correlations between offspring size (CLS) and offspring mass (ENM) of lizards.

	*r*	*p*
*Phrynocephalus axillaris*	0.042	0.882
*Phrynocephalus erythrurus*	−0.036	0.824
*Phrynocephalus forsythii*	−0.418	0.057
*Phrynocephalus grumgrzimailoi*	0.067	0.738
*Phrynocephalus helioscopus*	−0.783	0.427
*Phrynocephalus mystaceus*	−0.477	0.683
*Phrynocephalus przewalskii*	−0.796	0.107
*Phrynocephalus theobaldi*	−0.015	0.929
*Phrynocephalus versicolor*	−0.138	0.343
*Phrynocephalus vlangalii*	0.335	0.148

## 4 Discussion

Our study focuses on the correlation between reproductive characteristics and morphological traits/metabolic rates in several species of *Phrynocephalus* lizards in/around the Qinghai‐Tibetan Plateau. The results indicate that there were no significant differences in reproductive characteristics among *Phrynocephalus* lizards with different reproductive modes. In the clutch/litter mass analysis, the best‐fit model included metabolic rates, while snout‐vent length and head shape appeared in the secondary model. For the egg/neonate mass, the best model included snout‐vent length and limb length, whereas metabolic rates or snout‐vent length and head shape were included in two additional secondary models.

Previous studies have reported positive correlations between the hatchling size and maternal size in several species of reptiles (LeBlanc et al. 2014; Vieira et al. 2014). Our results showed a significant correlation between maternal SVL and CLM and ENM. This suggests that offspring mass is constrained by maternal size, which aligns with previous studies. The abdominal capacity of females is believed to be directly related to reproductive output (Scharf and Meiri 2013). On the contrary, there was no significant correlation between offspring number and SVL, suggesting that in this study, larger maternity allocated more energy and nutrients toward producing larger offspring rather than increasing their number. This phenomenon is more likely in species where hatchlings face fewer selective pressures, leading natural selection to favor optimizing offspring mass over number (Gatto et al. 2020). Larger offspring sizes usually have higher survival rates since they can store more fat for development, have a wider range of available prey items, and are less vulnerable to predators; hence, for offspring, it is advantageous to have larger body size at birth (Ford and Seigel 1989; Rivas and Burghardt 2001; Kissner and Weatherhead 2005). Our results are consistent with previous findings in other reptiles in that larger females tend to produce larger offspring (Nafus et al. 2015).

Some studies suggest that maternal behavior can also influence the number and size of offspring. Factors such as nest site selection, parental care, and predator avoidance (e.g., larger females, stronger hunting abilities, and predator avoidance skills) can provide a safe and nurturing environment with sufficient nutrition for newly hatched offspring (Mitchell et al. 2015). Our results show a significant correlation between offspring mass and limb length, head shape. Larger limb lengths could contribute to attaining a high sprint speed that should have fitness consequences as a way of escaping from predators (Tan et al. 2020) or be related to trophic ecology (Edwards et al. 2013). During gestation, both the thermal and nutritional environments chosen by the maternal organism significantly impact the offspring (Swain and Jones 2000). During the reproductive period, maternal organisms with elevated activity levels can better evade predators and secure additional prey to satisfy their increased energy requirements, enabling them to produce larger offspring (Bestion et al. 2022).

Our results of the partial correlation analysis found no significant trade‐off between offspring number and mass in females *Phrynocephalus* lizards. This affirms the results reported in both lizards ( *Lacerta vivipara* ) and snakes ( *Lycodon rufozonatus* ), where females do not trade off offspring mass against number (Uller and Olsson 2005; Guo et al. 2022). For ectotherms like reptiles, the total energy allocated to reproduction is positively correlated with maternal size, but there are variations in reproductive investment per offspring both within species and among species (Cooper and Vitt 1989; Shine et al. 1998; Shine 2005). Our study demonstrated a significant positive correlation between SMR and offspring mass. It generally suggests that individuals capable of maintaining a high metabolic rate may support a more active and productive way of life, allocating more resources to reproduction. However, after controlling for maternal body mass, metabolic rate showed no significant correlation with either offspring number or size, similar to the results of a previous meta‐analysis, which found nonsignificant correlations between reproduction and metabolic rates (Arnold et al. 2021).

According to previous studies, the transitions from oviparity to viviparity have occurred multiple times within squamate reptiles, and it is often associated with cooler climatic conditions where viviparity offers advantages over oviparity (Shine 1983; Feldman et al. 2015; Lawing et al. 2016). Females employ behavioral thermoregulation to maintain optimal temperatures for their uterine offspring, thereby enhancing their survival rates compared to those in colder external nests (Shine 1995). Compared with viviparous lizards, oviparous lizards are primarily distributed in low‐altitude regions, with higher temperatures, a greater availability of resources, and longer periods of activity and breeding time (Jin et al. 2018; Chen et al. 2022). Given the extent of these changes, other reproductive traits (such as offspring size and number) would be expected to change.

However, our results contradicted this expectation, indicating that reproductive mode does not significantly contribute to differences in reproductive output across genus *Phrynocephalus*. This divergence stemmed from the specific ecological conditions of the Qinghai‐Tibetan Plateau, where continuous geological activities over recent million years, along with factors such as altitude, temperature fluctuations, and resource availability, might impact reproductive traits differently than in other studied populations. Additionally, the evolutionary adaptations of *Phrynocephalus* lizards may have resulted in deviations from the expected patterns observed in other reptile species (Gao et al. 2019). Previous research on the relationship between reproductive modes and traits in reptiles has yielded conflicting findings, highlighting the complexity of factors that influence the evolution of energy investment and reproductive strategies. For example, Mesquita et al. (2016), Meiri et al. (2020) reported similar reproductive output between oviparous and viviparous lizards. Roitberg et al. (2013) found no difference in offspring size or number between oviparous and viviparous populations of *Zootoca vivipara*. However, Recknagel and Elmer (2019) reported that viviparous common lizards have lower reproductive output than their oviparous counterparts. Gao et al. (2019) suggested that in reptiles, most of the changes involved in the oviparity–viviparity transition were related to gene expression. Therefore, the occasional reversal from viviparity back to oviparity may not be as difficult as previously thought. In our study, the change in reproductive mode did not affect the number and mass of offspring, which may help explain why reproductive mode transitions are so frequent in squamate populations, as it suggests the shift can occur without a major immediate cost to offspring viability.

## 5 Conclusions

Our study reveals that *Phrynocephalus* lizards with larger snout‐vent lengths and higher standard metabolic rates tend to have heavier clutch/litter mass and egg/neonate mass. However, there were no noticeable changes in clutch/litter size. Our findings showed that the reproductive strategies of the *Phrynocephalus* lizards are inconsistent with the optimal offspring size theory. Furthermore, our results indicate that the transition between oviparity and viviparity in this genus does not significantly alter offspring number and mass, suggesting that such evolutionary shifts might occur without a major immediate cost to offspring viability. This finding provides an alternative perspective to the conventional understanding of reptilian life‐history evolution, particularly for species adapted to extreme environments like the Qinghai‐Tibetan Plateau. Based on our findings, future research should aim to validate these patterns through long‐term field studies that can assess the influence of natural environmental variables. Additionally, exploring the underlying genetic and endocrine mechanisms that regulate the allocation of resources would provide a more complete mechanistic understanding of this fascinating adaptive pathway.

## Author Contributions

**Xiaqiu Tao:** conceptualization (equal), data curation (equal), investigation (equal), resources (equal), software (lead), writing – original draft (equal). **Fan Xie:** data curation (equal), investigation (equal), writing – review and editing (equal). **Lin Zhu:** data curation (equal), investigation (equal), writing – review and editing (equal). **Qiannian Wu:** resources (equal), validation (equal), writing – review and editing (equal).

## Conflicts of Interest

The authors declare no conflicts of interest.

## Data Availability

The data that support the findings of this study are provided in the Dryad repository at (https://doi.org/10.5061/dryad.q83bk3jtd).
