# Correction to “Correlation of Morphology and Metabolism of Reproductive Traits in the Genus *Phrynocephalus* Around the Qinghai‐Tibetan Plateau”

**DOI:** 10.1002/ece3.72523

**Published:** 2025-11-14

Tao, X., F. Xie, L. Zhu, and Q. Wu. 2025. “Correlation of Morphology and Metabolism of Reproductive Traits in the Genus *Phrynocephalus* Around the Qinghai‐Tibetan Plateau.” *Ecology and Evolution* 15, no. 8: e72029

In Table 1 of the published article, the data for egg or neonate mass (ENM) and relative clutch or litter mass (RCLM) were inadvertently transcribed incorrectly: the ENM values were listed under the RCLM column, and the RCLM values were listed under the ENM column.

**TABLE 1:** Descriptive statistics, expressed as mean ± SE and range, for phenotype (SVL, AL, HL, HW, SL, SW, FLL, HLL), metabolic rate (SMR, mSMR) of newly hatched (oviparous species, O) and newborn (viviparous species, V) lizards.

Species	*N*	Mode	SVL	AL	HL	HW	SL	SW	FLL	HLL	CLS	CLM	ENM	RCLM	SMR	mSMR
			(mm)	(mm)	(mm)	(mm)	(mm)	(mm)	(mm)	(mm)		(g)	(g)			
*P. axillaris*	24	O	46.53 ± 0.56	22.35 ± 0.41	11.56 ± 0.17	10.63 ± 0.14	6.12 ± 0.11	9.80 ± 0.15	13.06 ± 0.26	18.48 ± 0.26	2.42 ± 0.22	1.14 ± 0.16	0.52 ± 0.03	0.34 ± 0.05	0.37 ± 0.06	0.11 ± 0.02
*P. erythrurus*	47	V	54.49 ± 0.45	28.04 ± 0.46	12.25 ± 0.09	10.99 ± 0.08	6.18 ± 0.08	10.04 ± 0.07	13.21 ± 0.15	17.28 ± 0.16	2.21 ± 0.11	1.79 ± 0.10	0.83 ± 0.02	0.34 ± 0.02	0.42 ± 0.05	0.08 ± 0.01
*P. forsythii*	32	V	47.41 ± 0.76	24.59 ± 0.53	11.14 ± 0.14	10.30 ± 0.11	5.45 ± 0.09	9.48 ± 0.11	12.55 ± 0.17	16.94 ± 0.28	3.56 ± 0.17	2.01 ± 0.12	0.58 ± 0.02	0.50 ± 0.04	0.48 ± 0.10	0.12 ± 0.02
*P. grumgrzimailoi*	39	O	54.02 ± 0.72	26.48 ± 0.52	13.09 ± 0.19	12.17 ± 0.18	6.78 ± 0.12	11.46 ± 0.29	15.11 ± 0.26	21.56 ± 0.26	2.18 ± 0.21	1.25 ± 0.19	0.62 ± 0.03	0.18 ± 0.03	0.78 ± 0.47	0.18 ± 0.13
*P. helioscopus*	8	O	46.03 ± 1.96	21.73 ± 1.11	11.70 ± 0.44	11.09 ± 0.38	6.21 ± 0.42	9.79 ± 0.55	14.12 ± 0.47	19.30 ± 0.39	3.00 ± 0.33	1.41 ± 0.12	0.46 ± 0.05	0.33 ± 0.07	0.87 ± 0.22	0.29 ± 0.08
*P. mystaceus*	5	O	75.38 ± 0.87	39.59 ± 1.15	19.06 ± 0.43	19.15 ± 0.28	11.71 ± 0.31	17.65 ± 0.38	25.85 ± 0.87	34.67 ± 0.79	3.00 ± 0.32	5.92 ± 0.62	1.82 ± 0.15	0.35 ± 0.07	1.13 ± 0.30	0.07 ± 0.02
*P. przewalskii*	9	O	51.57 ± 0.89	25.94 ± 0.48	13.26 ± 0.25	12.04 ± 0.23	6.82 ± 0.19	10.93 ± 0.25	14.77 ± 0.18	21.15 ± 0.21	1.22 ± 0.15	0.96 ± 0.10	0.75 ± 0.06	0.16 ± 0.02	—	—
*P. theobaldi*	41	V	47.58 ± 0.56	25.17 ± 0.41	11.44 ± 0.13	10.05 ± 0.10	6.02 ± 0.09	8.99 ± 0.10	12.56 ± 0.16	16.71 ± 0.18	2.32 ± 0.12	1.49 ± 0.10	0.65 ± 0.02	0.36 ± 0.03	0.29 ± 0.04	0.07 ± 0.01
*P. versicolor*	64	O	46.91 ± 0.35	22.76 ± 0.27	11.45 ± 0.09	10.53 ± 0.08	6.01 ± 0.06	9.58 ± 0.07	12.81 ± 0.14	18.34 ± 0.18	2.28 ± 0.15	1.12 ± 0.09	0.49 ± 0.02	0.30 ± 0.03	0.34 ± 0.06	0.11 ± 0.03
*P. vlangalii*	21	V	63.23 ± 0.91	34.23 ± 1.00	14.43 ± 0.18	12.51 ± 0.15	7.73 ± 0.13	11.08 ± 0.14	16.41 ± 0.21	21.77 ± 0.28	2.67 ± 0.21	2.54 ± 0.27	0.92 ± 0.05	0.20 ± 0.01	—	—

A corrected version of Table 1 is provided on the following page. No other parts of the article are affected, and this error does not alter the results or conclusions of the study.

We apologize for this error.

